# Fixed Point Theorems for Hybrid Mappings

**DOI:** 10.1155/2015/938165

**Published:** 2015-01-05

**Authors:** Maria Samreen, Tayyab Kamran, Erdal Karapinar

**Affiliations:** ^1^School of Natural Sciences, National University of Sciences and Technology, H-12, Islamabad 44000, Pakistan; ^2^Department of Mathematics, Quaid-i-Azam University, Islamabad 44000, Pakistan; ^3^Department of Mathematics, Incek, 06586 Ankara, Turkey; ^4^Nonlinear Analysis and Applied Mathematics Research Group (NAAM), King Abdulaziz University, Jeddah, Saudi Arabia

## Abstract

We obtain some fixed point theorems for two pairs of hybrid mappings using hybrid tangential property and quadratic type contractive condition. Our results generalize some results by Babu and Alemayehu and those contained therein. In the sequel, we introduce a new notion to generalize occasionally weak compatibility. Moreover, two concrete examples are established to illuminate the generality of our results.

## 1. Introduction and Preliminaries

Throughout this paper *X* is a metric space with metric *d*. For *x* ∈ *X* and *A*⊆*X*, *d*(*x*, *A*) = inf⁡{*d*(*x*, *y*) : *y* ∈ *A*}. We denote by CL(*X*) the class of all nonempty closed subsets of *X* and by CB(*X*) the class of all nonempty bounded closed subsets of *X*. For every *A*, *B* ∈ CL(*X*), let

(1) $$\begin{array}{c} H\left(A,B\right)=\left\{\begin{array}{cc} \max\left\{\underset{x\in A}{\sup}\;d\left(x,B\right),\underset{y\in B}{\sup}\;d\left(y,A\right)\right\}, & \\ \;\text{if}\;\text{the}\;\text{maximum}\;\text{exists} & \\ \infty , & \\ \;\text{otherwise}. & \end{array}\right. \end{array}$$

Such a map *H* is called generalized Hausdorff metric induced by *d*. Notice that *H* is a metric on CB(*X*). A point *p* ∈ *X* is said to be a fixed point of *T* : *X* → CL(*X*) if *p* ∈ *Tp*. The point *p* is called a coincidence point of *f* : *X* → *X* and *T* : *X* → CL(*X*) if *fp* ∈ *Tp*. The set of coincidence points of *f* and *T* is denoted by *C*(*f*, *T*). If *T* and *f* are both self-maps on *X*. The point *p* is called a coincidence point of *f* : *X* → *X* and *T* : *X* → *X* if *fp* = *Tp*. A pair (*f*, *T*) is known as hybrid pair where *f* : *X* → *X* and *T* : *X* → CL(*X*).

### 1.1. Compatibility and Property (*E*.*A*)

Sessa [1] introduced the concept of weakly commuting maps. Jungck [2] defined the notion of compatible maps in order to generalize the concept of weak commutativity and showed that weakly commuting maps are compatible but the converse is not true [2]. Pant [3–6] initiated the study of noncompatible maps. Sastry and Krishna Murthy [7] defined the notion of tangential single-valued maps. Aamri and El Moutawakil [8] rediscovered the notion of tangential maps and named it as property (*E*.*A*). The class of maps satisfying property (*E*.*A*) has remarkable property that it contains the class of compatible maps as well as the class of noncompatible maps [8]. Kamran [9] extended the notion of property (*E*.*A*) to a hybrid pair. Liu et al. [10] defined common property (*E*.*A*) for two hybrid pairs. Kamran and Cakic [13] introduced the hybrid tangential property and showed that it properly generalizes the notion of common property (*E*.*A*)  [22, Example 2.3]. In [11], the authors discussed fixed point theory problems in the context of *G*-metric space. Furthermore, in [11] the authors investigated the existence of a fixed point for multivalued mappings of integral type employing strongly tangential property (see also [12–16]).

For the sake of completeness, we recall some basic definitions and results.

**Definition 1 .**

Let *f* and *g* be self-maps on *X*. The pair (*f*, *g*) is said to

- be compatible [2] if lim⁡_*n*→*∞*_ *d*(*fgx*_*n*_, *gfx*_*n*_) = 0, whenever *x*_*n*_ is a sequence in *X* such that lim⁡_*n*→*∞*_ *fx*_*n*_ = lim⁡_*n*→*∞*_ *gx*_*n*_ = *t*, for some *t* ∈ *X*;
- be noncompatible if there is at least one sequence {*x*_*n*_} in *X* such that lim⁡_*n*→*∞*_ *fx*_*n*_ = lim⁡_*n*→*∞*_ *gx*_*n*_ = *t*, for some *t* ∈ *X*, but lim⁡_*n*→*∞*_ *d*(*fgx*_*n*_, *gfx*_*n*_) is either nonzero or nonexistent;
- satisfy property (*E*.*A*) [8] if there exists a sequence {*x*_*n*_} in *X* such that lim⁡_*n*→*∞*_ *fx*_*n*_ = lim⁡_*n*→*∞*_ *gx*_*n*_ = *t*, for some *t* ∈ *X*.

**Definition 2 .**

Let *f*, *g* be self-maps on *X* and let *T*, *S* be multivalued maps from *X* to CL(*X*).

- The maps *f* and *T* are said to be compatible [17] if *fTx* ∈ CL(*X*) for all *x* ∈ *X* and *H* (*fTx*_*n*_, *Tfx*_*n*_) → 0 whenever {*x*_*n*_} is a sequence in *X* such that *Tx*_*n*_ → *A* ∈ CL(*X*) and *fx*_*n*_ → *t* ∈ *A*.
- The maps *f* and *T* are noncompatible if *fTx* ∈ CL(*X*) for all *x* ∈ *X* and there exists at least one sequence {*x*_*n*_} in *X* such that *Tx*_*n*_ → *A* ∈ CL(*X*) and *fx*_*n*_ → *t* ∈ *A* but lim⁡_*n*→*∞*_ *H* (*fTx*_*n*_, *Tfx*_*n*_) ≠ 0 or is nonexistent.
- The maps *f* and *T* are said to satisfy property (*E*.*A*) [9] if there exists a sequence {*x*_*n*_} in *X*, some *t* ∈ *X*, and *A* ∈ CL(*X*) such that lim⁡_*n*→*∞*_ *fx*_*n*_ = *t* ∈ *A* = lim⁡_*n*→*∞*_ *Tx*_*n*_.
- The hybrid pairs (*f*, *T*) and (*g*, *S*) are said to satisfy common property (*E*.*A*) [10] if there exist two sequences {*x*_*n*_}, {*y*_*n*_} in *X*, some *t* ∈ *X*, and *A*, *B* ∈ CB(*X*) such that lim⁡_*n*→*∞*_ *Tx*_*n*_ = *A*, lim⁡_*n*→*∞*_ *Sy*_*n*_ = *B*, and lim⁡_*n*→*∞*_ *fx*_*n*_ = lim⁡_*n*→*∞*_ *gy*_*n*_ = *t* ∈ *A*∩*B*.
- The hybrid pair (*f*, *T*) is said to be *g*-*tangential* at *t* ∈ *X* [13] if there exist two sequences {*x*_*n*_}, {*y*_*n*_} in *X*, *A* ∈ CL(*X*) such that lim⁡_*n*→*∞*_ *Sy*_*n*_ ∈ CL(*X*) and lim⁡_*n*→*∞*_ *fx*_*n*_ = lim⁡_*n*→*∞*_ *gy*_*n*_ = *t* ∈ *A* = lim⁡_*n*→*∞*_ *Tx*_*n*_.

### 1.2. Weak Compatibility and Weak Commutativity

Jungck [18] introduced the notion of weak compatibility and in [19] Jungck and Rhoades further extended weak compatibility to a hybrid pair of single-valued and multivalued maps. Singh and Mishra [20] introduced the notion of (*IT*)-commutativity for a hybrid pair to generalize the notion of weak compatibility. Kamran [9] introduced the notion of *T*-weak commutativity and showed that (*IT*)-commutativity implies *T*-weak commutativity but the converse is not true in general [9, Example 3.8]. Al-Thagafi and Shahzad [21] introduced the class of occasionally weakly compatible single-valued maps and showed that the weakly compatible maps form a proper subclass of the occasionally weakly compatible maps [21, Example]. Abbas and Rhoades [23] generalized the notion of occasionally weak compatibility to a hybrid pair.

**Definition 3 .**

Let *f* and *g* be self-maps on *X*. The pair (*f*, *g*) is said to

(iv) be weakly compatible [18] if *fgx* = *gfx* whenever *fx* = *gx*, *x* ∈ *X*;
(v) be occasionally weakly compatible (owc) [21] if *fgx* = *gfx* for some *x* ∈ *C*(*f*, *g*).

**Definition 4 .**

Let *f* be a self-map on *X* and *T* from *X* to CL(*X*).

- The maps *f* and *T* are weakly compatible [19] if they commute at their coincidence points, that is, *fTx* = *Tfx* whenever *fx* ∈ *Tx*.
- The maps *f* and *T* are said to be (*IT*)-commuting [20, 22] at *x* ∈ *X* if *fTx*⊆*Tfx*.
- The map *f* is said to be *T*-weakly [9] commuting at *x* ∈ *X* if *ffx* ∈ *Tfx*.
- The maps *f* and *T* are said to be occasionally weakly compatible [23] if and only if there exists some point *x* ∈ *X* such that *fx* ∈ *Tx* and *fTx*⊆*Tfx*.

Recently, Babu and Alemayehu [24] obtained some fixed point theorems for single-valued mappings using property (*E*.*A*), common property (*E*.*A*), and occasionally weak compatibility. The purpose of this paper is to extend the main results of [24] to hybrid pairs. We also introduce a new notion for a hybrid pair that generalizes occasionally weak compatibility.

## 2. Main Results

We begin with the following proposition.

**Proposition 5 .**

Let (*X*, *d*) be a metric space, let *f*, *g* be self-maps on *X*, and let *S*, *T* be mappings from *X* to *CL*(*X*) such that

(2) HTx,Sy2 ≤c1max⁡{dfx,Tx2,dgy,Sy2,dfx,gy2}  +c2max⁡⁡dfx,Txdfx,Sy,dgy,Sydgy,Tx  +c3d(fx,Sy)d(gy,Tx)

for all *x*, *y* ∈ *X*, where *c*_1_, *c*_2_, *c*_3_ ≥ 0 and *c*_1_ < 1. Suppose that either

- *TX*⊆*gX*, the pair (*f*, *T*) satisfies property (*E*.*A*) and *fX* is closed subspace of *X*, or
- *SX*⊆*fX*, the pair (*g*, *S*) satisfies property (*E*.*A*) and *gX* is closed subspace of *X*.

Then *C*(*f*, *T*) ≠ *∅* and *C*(*g*, *S*) ≠ *∅*.

**Proof**

Suppose that (I) holds; then there exists a sequence {*x*_*n*_} in *X* and *A* ∈ CL(*X*) such that

(3) $$\begin{array}{c} \underset{n\to \infty }{\lim}fx_{n}=z\in A=\underset{n\to \infty }{\lim}Tx_{n}. \end{array}$$

Since *TX*⊆*gX* then *Tx*_*n*_⊆*gX* for all *n*. Now for *z* ∈ *A* we have

(4) $$\begin{array}{c} d\left(z,gX\right)\leq d\left(z,Tx_{n}\right)\;\forall n. \end{array}$$

Now by using the definition of Hausdorff metric, we have

(5) $$\begin{array}{c} d\left(z,gX\right)\leq d\left(z,Tx_{n}\right)\leq H\left(A,Tx_{n}\right)\;\forall n. \end{array}$$

Applying limit throughout we have

(6) $$\begin{array}{c} d\left(z,gX\right)\leq \underset{n\to \infty }{\lim}d\left(x,Tx_{n}\right)\leq \underset{n\to \infty }{\lim}H\left(A,Tx_{n}\right)=0, \end{array}$$

which infers that $z\in \bar{gX}$. Therefore, there exists a sequence {*y*_*n*_} in *X* such that lim⁡_*n*→*∞*_ *gy*_*n*_ = *z*. Consider the following:

(7) $$\begin{array}{c} \underset{n\to \infty }{\lim}fx_{n}=\underset{n\to \infty }{\lim}gy_{n}=z. \end{array}$$

Since *fX* is closed, there exists *a* ∈ *X* such that

(8) $$\begin{array}{c} \underset{n\to \infty }{\lim}fx_{n}=fa=z. \end{array}$$

We claim that lim⁡_*n*→*∞*_ *Sy*_*n*_ = *A*. From (25) we get

(9) HTxn,Syn2 ≤c1max⁡dfxn,Txn2,dgyn,Syn2,hhhhhhhhhhdfxn,gyn2  +c2max⁡⁡d(fxn,Txn)d(fxn,Syn),hhhhhhhhhhhhd(gyn,Syn)d(gyn,Txn)  +c3d(fxn,Syn)d(gyn,Txn).

Using (3) and (7) we get

(10) limsup⁡n→∞HA,Syn2≤c1limsup⁡n→∞dz,Syn2≤c1limsup⁡n→∞HA,Syn2.

Since *c*_1_ < 1, it follows that lim⁡_*n*→*∞*_ *H*(*A*, *Sy*_*n*_) = 0 and hence

(11) $$\begin{array}{c} \underset{n\to \infty }{\lim}Sy_{n}=A. \end{array}$$

Now we show that *a* ∈ *C*(*f*, *T*). Using (25) we have

(12) HTa,Syn2 ≤c1max⁡⁡{dfa,Ta2,dgyn,Syn2,dfa,gyn2}  +c2max⁡dfa,Tadfa,Syn,hhhhhhhhhhhhd(gyn,Syn)d(gyn,Ta)  +c3d(fa,Syn)d(gyn,Ta).

Letting *n* → *∞* and using (3), (7), (8), (11), and definition of Hausdorff metric the above inequality yields

(13) $$\begin{array}{c} d{\left[\left(fa,Ta\right)\right]}^{2}\leq {\left[H\left(A,Ta\right)\right]}^{2}\leq c_{1}d{\left[\left(fa,Ta\right)\right]}^{2}. \end{array}$$

Since *c*_1_ < 1, using closedness of *Ta*, it follows that

(14) $$\begin{array}{c} fa\in Ta. \end{array}$$

Since *TX*⊆*gX*, there exists *b* ∈ *X* such that

(15) $$\begin{array}{c} gb=fa. \end{array}$$

Now we show that *b* ∈ *C*(*g*, *S*); from (25), (14), and (15) we have

(16) dgb,Sb2 =dfa,Sb2 ≤HTa,Sb2 ≤c1max⁡⁡{dfa,Ta2,dgb,Sb2,dfa,gb2}  +c2max⁡{d(fa,Ta)d(fa,Sb),d(gb,Sb)d(gb,Ta)}  +c3d(fa,Sb)d(gb,Ta) ≤c1[dgb,Sb2].

Since *c*_1_ < 1, closedness of *Sb* implies *gb* ∈ *Sb*. Similarly, the assertion of proposition holds if we assume (II).

**Remark 6 .**

Note that if *T* is a self-map on *X*, Proposition 5 reduces to [24, Proposition 2.1].

Now we introduce the notion of occasionally weak commutativity.

**Definition 7 .**

Let (*f*, *T*) be a hybrid pair. The mapping *f* is said to be occasionally *T*-weakly commuting if and only if there exists some *x* ∈ *X* such that *fx* ∈ *Tx* and *ffx* ∈ *Tfx*.

Note that if a hybrid pair (*f*, *T*) is occasionally weakly compatible at *x* ∈ *X* then *f* is occasionally *T*-weakly commuting at *x*. The following example shows that the converse of the above statement is not true.

**Example 8 .**

Let *X* = [1, *∞*) with the usual metric. Define *f* : *X* → *X*, *T* : *X* → CL(*X*) by *fx* = 2*x* and *Tx* = [1,2*x* + 1] for all *x* ∈ *X*. Then for all *x* ∈ *X*, *fx* ∈ *Tx*, *ffx* = 4*x* ∈ [1,4*x* + 1] = *Tfx*, and fTx=[2,4x+2] ⫅ Tfx. Therefore *f* is occasionally weakly compatible at any *x* ∈ *X*.

Our next result extends [24, Theorem 2.2] to hybrid pairs. Note that in the hypothesis of our result we assumed that hybrid pairs satisfy occasionally weak commutativity rather than using the notion of occasionally weak compatibility.

**Theorem 9 .**

In addition to the hypothesis of Proposition 5 on *f*, *g*, *S*, and *T*,

- if *f* is occasionally *T*-weakly commuting at *a* and *ffa* = *fa* then *f* and *T* have a common fixed point;
- if *g* is occasionally *S*-weakly commuting at *b* and *ggb* = *gb* then *g* and *S* have a common fixed point;
- *f*, *g*, *S*, and *T* have a common fixed point if both (i) and (ii) hold.

**Proof**

By (i), we have *ffa* = *fa* and *ffa* ∈ *Tfa*. Thus *z* = *fz* ∈ *Tz*. This proves (i). (ii) can be proved on the same lines; then (iii) is immediately followed.

**Example 10 .**

Let *X* = [1/4, 1) with the usual metric. Define mappings *f*, *g* : *X* → *X* and *T*, *S* : *X* → CL(*X*) by

(17) $$\begin{array}{c} fx=\left\{\begin{array}{cc} \frac{2}{3} & \text{if}\;\;\frac{1}{4}\leq x<\frac{3}{4}\\ 1-\frac{x}{3} & \text{if}\;\;\frac{3}{4}\leq x<1, \end{array}\right.\\ gx=\left\{\begin{array}{cc} \frac{2}{3} & \text{if}\;\;\frac{1}{4}\leq x<\frac{3}{4}\\ \frac{1}{2}+\frac{x}{3} & \text{if}\;\;\frac{3}{4}\leq x<1, \end{array}\right.\\ Tx=\left\{\begin{array}{cc} \left\{\frac{3}{4}\right\} & \text{if}\;\;\frac{1}{4}\leq x<\frac{3}{4}\\ \left[\frac{3}{4},\frac{4}{5}\right] & \text{if}\;\;\frac{3}{4}\leq x<1, \end{array}\right.\\ Sx=\left\{\begin{array}{cc} \left[\frac{4}{5},\frac{5}{6}\right] & \text{if}\;\;\frac{1}{4}\leq x<\frac{3}{4}\\ \left[\frac{3}{4},\frac{4}{5}\right] & \text{if}\;\;\frac{3}{4}\leq x<1. \end{array}\right. \end{array}$$

We observe that *TX*⊆*gX*, *fX* is closed, and *gX* is open; neither *SX*⊆*gX* nor *gX*⊆*SX*. There exists a sequence {*x*_*n*_}; *x*_*n*_ = 3/4 + 1/*n*, *n* = 5,6, 7,…   in *X* with lim⁡_*n*→*∞*_ *fx*_*n*_ = 3/4 ∈ lim⁡_*n*→*∞*_ *Tx*_*n*_, so that the hybrid pair (*f*, *T*) satisfies property (*E*.*A*) but it is not compatible. Inequality (25) is satisfied for *c*_1_ = 1/2 < 1, *c*_2_ = 2, and *c*_3_ = 0. Also note that *f* is occasionally *T*-weakly commuting at point 3/4 and *g* is occasionally *S*-weakly commuting at each point in the interval [3/4, 9/10]. Furthermore (i), (ii), and (iii) of Theorem 9 are also satisfied at point 3/4. Hence *f*, *g*, *S*, and *T* have common fixed point 3/4.

In the next result we will use the notion of hybrid tangential property and occasionally weak commutativity to extend and improve [24, Proposition 2.5].

**Theorem 11 .**

Let (*X*, *d*) be a metric space, let *f*, *g* be self-maps on *X*, and let *S*, *T* be mappings from *X* to *CL*(*X*) satisfying inequality (25). Assume *fX*, *gX* are closed subspaces of X and further suppose that either

- (*f*, *T*) is *g*-tangential or
- (*g*, *S*) is *f*-tangential.

Then *C*(*f*, *T*) ≠ *∅* and *C*(*g*, *S*) ≠ *∅*. Furthermore,

- if *f* is occasionally *T*-weakly commuting at *a* and *ffa* = *fa* then *f* and *T* have a common fixed point;
- if *g* is occasionally *S*-weakly commuting at *b* and *ggb* = *gb* then *g* and *S* have a common fixed point;
- *f*, *g*, *S*, and *T* have a common fixed point if both (i) and (ii) hold.

**Proof**

Suppose that hybrid pair (*f*, *T*) is *g*-tangential; then there exist sequences *x*_*n*_ and *y*_*n*_ in *X* such that

(18) $$\begin{array}{c} \underset{n\to \infty }{\lim}fx_{n}=\underset{n\to \infty }{\lim}gy_{n}=t\in A=\underset{n\to \infty }{\lim}Tx_{n},\\ \underset{n\to \infty }{\lim}Sy_{n}=B\in \text{CL}\left(X\right). \end{array}$$

Now we prove that *A* = *B*; from (25) we have

(19) HTxn,Syn2 ≤c1max⁡dfxn,Txn2,dgyn,Syn2,⁡hhhhhhhhhhhdfxn,gyn2  +c2max⁡dfxn,Txndfxn,Syn,hhhhhhhhhhhidgyn,Syndgyn,Txn  +c3dfxn,Syndgyn,Txn.

On taking limit *n* → *∞* and using (18), we get

(20) $$\begin{array}{c} {\left[H\left(A,B\right)\right]}^{2}\leq c_{1}{\left[d\left(t,B\right)\right]}^{2}\leq c_{1}{\left[H\left(A,B\right)\right]}^{2}, \end{array}$$

which implies [*H*(*A*, *B*)] = 0; hence *A* = *B*. Since *fX* and *gX* are closed there exists *a*, *b* ∈ *X* such that

(21) $$\begin{array}{c} \underset{n\to \infty }{\lim}fx_{n}=fa=t=gb=\underset{n\to \infty }{\lim}gy_{n}. \end{array}$$

The rest of the proof runs on the same lines as that of Proposition 5 and Theorem 9.

**Corollary 12 .**

Let (*X*, *d*) be a metric space, let *f*, *g* be self-maps on *X* and *S*, and let *T* be mappings from *X* to *CL*(*X*) satisfying inequality (25) of Proposition 5. Suppose that pairs (*f*, *T*)  (*g*, *S*) satisfy common property (*E*.*A*) and *fX*, *gX* are closed subsets of X; then *C*(*f*, *T*) ≠ *∅* and *C*(*g*, *S*) ≠ *∅*. Furthermore,

- if *f* is occasionally *T*-weakly commuting at *a* and *ffa* = *fa* then *f* and *T* have a common fixed point;
- if *g* is occasionally *S*-weakly commuting at *b* and *ggb* = *gb* then *g* and *S* have a common fixed point;
- *f*, *g*, *S*, and *T* have a common fixed point if both (i) and (ii) hold.

**Remark 13 .**

If *S* and *T* are self-maps on *X* then Corollary 12 coincides with [24, Proposition 2.5].

**Example 14 .**

Let *X* = [1/4, 1) with the usual metric. Define mappings *f*, *g* : *X* → *X* and *T*, *S* : *X* → CL(*X*) by

(22) $$\begin{array}{c} fx=\left\{\begin{array}{cc} \frac{2}{3} & \text{if}\;\;\frac{1}{4}\leq x<\frac{3}{4}\\ 1-\frac{x}{3} & \text{if}\;\;\frac{3}{4}\leq x<1, \end{array}\right.\\ gx=\left\{\begin{array}{cc} \frac{5}{6} & \text{if}\;\;\frac{1}{4}\leq x<\frac{3}{4}\\ \frac{1}{2}+\frac{x}{3} & \text{if}\;\;\frac{3}{4}\leq x<1, \end{array}\right.\\ Tx=\left\{\begin{array}{cc} \left[\frac{1}{4},\frac{1}{3}\right] & \text{if}\;\;\frac{1}{4}\leq x<\frac{3}{4}\\ \left[\frac{3}{4},\frac{4}{5}\right] & \text{if}\;\;\frac{3}{4}\leq x<1, \end{array}\right.\\ Sx=\left\{\begin{array}{cc} \left[\frac{2}{3},\frac{3}{4}\right] & \text{if}\;\;\frac{1}{4}\leq x<\frac{3}{4}\\ \left[\frac{3}{4},\frac{4}{5}\right] & \text{if}\;\;\frac{3}{4}\leq x<1. \end{array}\right. \end{array}$$

In this example *fX* and *gX* are closed subspaces of *X*; neither *SX*⊆*fX* nor *TX*⊆*gX*. There exists a sequence {*x*_*n*_}; *x*_*n*_ = 3/4 + 1/*n*, *n* = 5,6, 7,… in *X* with lim⁡_*n*→*∞*_ *fx*_*n*_ = lim⁡_*n*→*∞*_ *gx*_*n*_ = 3/4 ∈ [3/4, 4/5], where lim⁡_*n*→*∞*_ *Tx*_*n*_ = lim⁡_*n*→*∞*_ *Sx*_*n*_ = [3/4, 4/5]. Hence (*f*, *T*) and (*g*, *S*) satisfy common property (*E*.*A*). It can be easily shown that the hybrid pairs (*f*, *T*) and (*g*, *S*) satisfy inequality (25) with *c*_1_ = 7/8, *c*_2_ = 6, and *c*_3_ = 0. Furthermore, *f* is occasionally *T*-weakly commuting at point 3/4 while *g* is occasionally *S*-weakly commuting at each point in the interval [3/4, 9/10]. Conditions (i), (ii), and (iii) of Corollary 12 hold true for *x* = 3/4; so *f*, *g*, *S*, and *T* have common fixed point 3/4.

In the following we include some of the consequences of Theorem 9.

**Corollary 15 .**

Let (*X*, *d*) be a metric space, let *f*, *g* be self-maps on *X*, and let *T* be mappings from *X* to *CL*(*X*) such that

(23) HTx,Ty2 ≤c1max⁡{dfx,Tx2,dgy,Ty2,dfx,gy2}  +c2max⁡{d(fx,Tx)d(fx,Ty),d(gy,Ty)d(gy,Tx)}  +c3d(fx,Ty)d(gy,Tx)

for all *x*, *y* ∈ *X*, where *c*_1_, *c*_2_, *c*_3_ ≥ 0 and *c*_1_ < 1. Suppose that either

- *TX*⊆*gX*, the pair (*f*, *T*) satisfies property (*E*.*A*) and *fX* is closed subspace of *X*, or
- *TX*⊆*fX*, the pair (*g*, *T*) satisfies property (*E*.*A*) and *gX* is closed subspace of *X*.

Then *C*(*f*, *T*) ≠ *∅* and *C*(*g*, *T*) ≠ *∅*. Furthermore

- if *f* is occasionally *T*-weakly commuting at *a* and *ffa* = *fa* then *f* and *T* have a common fixed point;
- if *g* is occasionally *T*-weakly commuting at *b* and *ggb* = *gb* then *g* and *T* have a common fixed point;
- *f*, *g*, and *T* have a common fixed point if both (i) and (ii) hold.

**Proof**

Take *S* = *T* in Theorem 9.

**Corollary 16 .**

Let (*X*, *d*) be a metric space, let *f* be a self-map on *X*, and let *T* be a mapping from *X* to *CL*(*X*) such that

(24) HTx,Ty2 ≤c1max⁡{dfx,Tx2,dfy,Ty2,dfx,fy2}  +c2max⁡{d(fx,Tx)d(fx,Ty),d(fy,Ty)d(fy,Tx)}  +c3d(fx,Ty)d(fy,Tx)

for all *x*, *y* ∈ *X*, where *c*_1_, *c*_2_, *c*_3_ ≥ 0 and *c*_1_ < 1. Suppose that *TX*⊆*fX*: the pair (*f*, *T*) satisfies property (*E*.*A*) and *fX* is closed subspace of *X*. Then *C*(*f*, *T*) ≠ *∅*. Furthermore if *f* is occasionally *T*-weakly commuting at *a* and *ffa* = *fa* then *f* and *T* have a common fixed point.

**Proof**

Take *S* = *T* and *g* = *f* in Theorem 9.

**Corollary 17 .**

Let (*X*, *d*) be a metric space, let *f* be a self-map on *X*, and let *T* be a mapping from *X* to *CL*(*X*) such that

(25) HTx,Ty2 ≤c1max⁡{dx,Tx2,dy,Ty2,dx,y2}  +c2max⁡{d(x,Tx)d(x,Ty),d(y,Ty)d(y,Tx)}  +c3d(x,Ty)d(y,Tx)

for all *x*, *y* ∈ *X*, where *c*_1_, *c*_2_, *c*_3_ ≥ 0 and *c*_1_ < 1. Suppose *X* is closed and the pair (*I*, *T*) satisfies property (*E*.*A*), where *I* is an identity map on *X*. Then *T* has a fixed point.

**Proof**

Take *S* = *T* and *g* = *f* = *I* in Theorem 9.

**Corollary 18 (see [24, Theorem 2.2]).**

Let *f*, *g*, *T*, and *S* be four self-maps on a complete metric space (*X*, *d*) satisfying the inequality

(26) dTx,Sy2 ≤c1max⁡{dfx,Tx2,dgy,Sy2,dfx,gy2}  +c2max⁡{d(fx,Tx)d(fx,Sy),d(gy,Sy)d(gy,Tx)}  +c3d(fx,Sy)d(gy,Tx)

for all *x*, *y* ∈ *X*, where *c*_1_, *c*_2_, *c*_3_ ≥ 0 and *c*_1_ + *c*_3_ < 1. Suppose that either

- *SX*⊆*fX*, the pair (*S*, *g*) satisfies property (*E*.*A*) and *gX* is closed subspace of *X*, or
- *TX*⊆*gX*, the pair (*T*, *f*) satisfies property (*E*.*A*) and *fX* is closed subspace of *X*, holds.

Then *C*(*f*, *T*) ≠ *∅* and *C*(*g*, *S*) ≠ *∅*. Furthermore if both the pairs (*f*, *T*) and (*g*, *S*) are occasionally weakly compatible on *X*, then the maps *f*, *g*, *T*, and *S* have a unique common fixed point in *X*.

**Proof**

Take *S*, *T* : *X* → *X* and *g*, *f* : *X* → *X* in Theorem 9. Moreover, uniqueness of fixed point is followed from inequality (26) as *c*_1_ + *c*_3_ < 1.

**Corollary 19 .**

Let (*X*, *d*) be a metric space and let *f*, *T* be self-maps on *X* such that

(27) dTx,Ty2 ≤c1max⁡{dfx,Tx2,dfy,Ty2,dfx,fy2}  +c2max⁡{d(fx,Tx)d(fx,Ty),d(fy,Ty)d(fy,Tx)}  +c3d(fx,Ty)d(fy,Tx)

for all *x*, *y* ∈ *X*, where *c*_1_, *c*_2_, *c*_3_ ≥ 0 and *c*_1_ + *c*_3_ < 1. Suppose that *TX*⊆*fX*: the pair (*f*, *T*) satisfies property (*E*.*A*) and *fX* is closed subspace of *X*. Then *C*(*f*, *T*) ≠ *∅*. Furthermore if the pair (*f*, *T*) is occasionally weakly compatible, then *f* and *T* have a unique common fixed point.

**Proof**

Take *S* = *T* : *X* → *X* and *g* = *f* in Theorem 9. Moreover, uniqueness of fixed point is followed from inequality (27) as *c*_1_ + *c*_3_ < 1.
